# Depressive Symptoms, Anxiety, Insomnia, and Sexual Quality of Life in Patients with Chronic Obstructive Pulmonary Disease from the Podlaskie Voivodeship, Poland: A Cross-Sectional Pilot Study

**DOI:** 10.3390/jcm15072769

**Published:** 2026-04-06

**Authors:** Katarzyna Bojarska, Magda Orzechowska, Mateusz Grochowski, Roman Skiepko, Mateusz Cybulski

**Affiliations:** 1 Department of Integrated Medical Care, Medical University of Bialystok, 15-089 Bialystok, Poland; 2 Department of Allergology and Internal Medicine, Medical University of Bialystok, 15-276 Bialystok, Poland

**Keywords:** anxiety, depression, chronic obstructive pulmonary disease, insomnia, mental health, sexual quality of life

## Abstract

**Introduction:** Chronic obstructive pulmonary disease (COPD) is associated with substantial symptom burden and functional limitations, which may co-occur with psychological distress. This pilot study aimed to assess depressive symptoms, anxiety, insomnia, and sexual quality of life in patients with COPD living in the Podlaskie Voivodeship. **Materials and Methods:** This cross-sectional pilot study included 47 patients with COPD, including outpatients (*n* = 11) and inpatients (*n* = 36), recruited at the University Teaching Hospital in Bialystok between February and August 2025. The original survey questionnaire, Beck Depression Inventory (BDI), Hamilton Anxiety Rating Scale (HAM-A), Generalized Anxiety Disorder-7 (GAD-7), Athens Insomnia Scale (AIS), Insomnia Severity Index (ISI), and Sexual Quality of Life (SQoL) questionnaires were used. **Results:** In the study sample, median scores indicated a considerable burden of depressive symptoms (BDI Me = 16), anxiety (HAM-A Me = 27; GAD-7 Me = 15), and insomnia (AIS Me = 9; ISI Me = 14), alongside reduced sexual quality of life (SQoL Me = 46). Age in the total sample correlated positively with depressive symptoms, anxiety, and sleep difficulties, and negatively with SQoL; however, these relationships were not consistently maintained in age-stratified analyses. Crude inpatient–outpatient differences were substantial, but supplementary adjusted models showed that subjective symptom severity was the most consistent predictor across outcomes, whereas the independent role of hospitalization status was attenuated. Strong associations were observed between depression, anxiety, insomnia, and sexual quality of life. **Conclusions:** This pilot study indicates a substantial within-sample psychological burden in patients with COPD and suggests that these outcomes are closely associated with subjective symptom burden. Given the small sample size, marked group imbalance, cross-sectional design, and lack of objective COPD severity measures, the findings should be interpreted as exploratory and require confirmation in larger multicenter studies.

## 1. Introduction

Chronic obstructive pulmonary disease (COPD) is a growing public health concern, especially in the context of an aging population and long-term exposure to environmental factors such as air pollution and tobacco smoke. Globally, COPD affects approximately 328 million people, representing 4.8% of the population; nearly 2 million patients suffer from it in Poland [1,2]. At present, this disease is the third leading cause of death worldwide, generating a significant social and economic burden [3].

Chronic symptoms characterizing COPD include, among others, shortness of breath, cough, limited effort tolerance, and frequent exacerbations requiring hospitalization—significantly reducing the quality of life of patients. Progressive physical limitations contribute to disengagement from professional and social activities, leading to isolation, increased dependence on caregivers, and impaired mental functioning [4,5,6]. Sleep disorders, frequently resulting from the nocturnal exacerbation of symptoms, further intensify exhaustion and reduce patients’ well-being [7,8].

COPD is currently regarded not only as a respiratory disorder, but also as a condition with significant systemic consequences, including adverse effects on mental functioning and patients’ quality of life [9,10,11]. Increasing evidence suggests that the coexistence of depressive symptoms, anxiety, and sleep disturbances in this population may result from complex biological mechanisms. Particular importance has been attributed to persistent systemic inflammation, which, through the activation of proinflammatory mediators, may modulate central nervous system activity, affect neurotransmission, and contribute to the development of psychiatric symptoms [9,11]. Chronic hypoxia also appears to play a substantial role, leading to impaired neuronal function, the deterioration of sleep architecture, and an increased psychological burden [10]. The pathophysiology of these associations further involves neuroimmune dysregulation, reflecting disturbed interactions among the immune system, the central nervous system, and stress response mechanisms, as well as autonomic imbalance, which may additionally exacerbate both somatic and emotional symptoms. Consequently, COPD should be considered a disease in which inflammatory, hypoxemic, and neuroregulatory mechanisms may jointly contribute to the development and persistence of psychiatric symptoms [9,10,11]. Mental health plays a crucial role in the disease course—depressive and anxiety symptoms tend to limit motivation to adhere to treatment recommendations, reduce physical activity, and may contribute to more frequent exacerbations further to being associated with an increased risk of death [12,13].

Sexuality is an important, yet still underrecognized, dimension of quality of life in patients with COPD. Importantly, the negative effect of COPD on sexual health is not explained solely by dyspnea, fatigue, weakness, and reduced exercise tolerance, but appears to result from interacting respiratory, autonomic, vascular–endocrine, and treatment-related mechanisms. COPD has been associated with autonomic dysfunction, including impaired heart rate variability and a relative predominance of sympathetic activity; recurrent hypoxemia and hypercapnia, increased respiratory effort, systemic inflammation, and β-sympathomimetic therapy may all contribute to this imbalance [14,15,16]. Because genital arousal, erection/vaginal lubrication, and orgasm depend on coordinated autonomic pathways, dysautonomia may translate into impaired desire, arousal, and sexual performance [17]. In parallel, hypoxemia may further worsen sexual health through reduced oxygen delivery and endocrine disturbances: in COPD, lower oxygen saturation has been associated with more severe erectile dysfunction, while severe hypoxemia has also been linked to lower testosterone concentrations, which may contribute to reduced libido [18,19]. Treatment-related factors should likewise be considered, as systemic glucocorticoid exposure in particular may suppress the hypothalamic–pituitary–gonadal axis and lower testosterone, thereby aggravating sexual symptoms [20]. Thus, sexual dysfunction in COPD should be viewed as a multifactorial consequence of respiratory limitation, autonomic dysregulation, hypoxemia-related vascular and endocrine changes, and the effects of treatment, rather than as an isolated consequence of exertional breathlessness alone.

Despite the growing awareness of the importance of mental health in the course of COPD, in many regions—including Poland—research specific to local populations is still scarce. The Podlaskie Voivodeship, characterized by smaller urbanization, a dispersed settlement structure, and an above-average percentage of elderly people, is an area where the issue in question may be particularly significant. Data on the mental health of COPD patients in this region are limited, hindering the organization of targeted preventive and therapeutic interventions.

Hence, the aim of this pilot study was to assess depressive symptoms, anxiety, insomnia, and sexual quality of life in patients with COPD living in the Podlaskie Voivodeship, and to examine their associations with selected socio-demographic and disease-related factors available in the study dataset.

The following research questions were addressed:

1. What is the burden of depressive symptoms, anxiety, and insomnia in the study sample of patients with COPD?
2. Are depressive symptoms, anxiety, insomnia, and sexual quality of life associated with age, sex, disease duration, subjective symptom severity, and treatment setting?
3. Are depression, anxiety, insomnia, and sexual quality of life interrelated in this sample?

## 2. Materials and Methods

### 2.1. Participants

The study involved outpatients of the Allergy Clinic of the University Teaching Hospital in Bialystok and inpatients of the Department of Allergy and Internal Medicine of the University Teaching Hospital in Bialystok diagnosed with COPD. Residents of the Podlaskie Voivodeship were eligible for the study. Between February and August of 2025, a total of 58 completed surveys were collected, of which 11 were rejected due to incomplete filling out. The final analysis concerned 47 patients, including 11 outpatients (4 women and 7 men) and 36 inpatients (15 women and 21 men). The participants ranged in age from 48 to 85 years, with a mean age of 66.87 years, median of 69 years, and interquartile range of 59–73 years. For supplementary analyses, age was additionally stratified into three categories: 18–59 years, 60–69 years, and ≥70 years.

As this was a pilot study, the final sample was intended to provide preliminary estimates of variability and associations rather than confirmatory evidence. A post hoc sensitivity assessment indicated that the present sample was primarily informative for moderate-to-large effects, and therefore the findings should be interpreted as exploratory.

**Figure 1:**
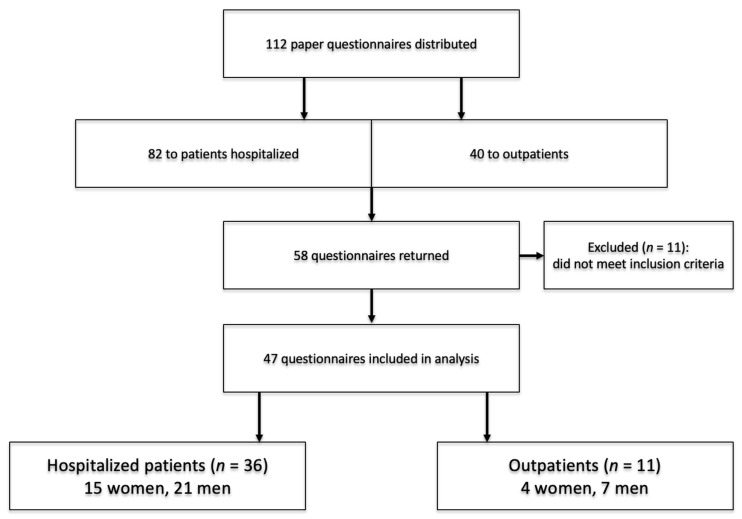
Scheme of participant selection for the study.

The participant selection process is presented in Figure 1.

### 2.2. Study Design

The study was cross-sectional in nature with a diagnostic survey approach used. Data were collected through self-report questionnaires completed by patients under the supervision of medical staff. The study was conducted under outpatient and inpatient conditions. Patients were notified of the study’s objectives, implementation, and voluntary nature. After providing informed consent, the participants completed the questionnaires in the presence of the research team.

This pilot study was designed to provide a preliminary evaluation of the mental health of patients with COPD living in the Podlaskie Voivodeship as well as to assess the feasibility of the research protocol. The pilot study enabled:

1. The assessment of the usefulness of the psychometric tools used in the investigated population;
2. The verification of data collection procedures under outpatient and inpatient conditions;
3. The identification of potential organizational and methodological challenges;
4. An estimation of the variability of the obtained results as the basis for designing a full-scale study.

Patients meeting the following criteria were included in the study:

1. Diagnosis of chronic obstructive pulmonary disease (COPD) confirmed in medical records (a diagnosis of COPD was accepted when a prior diagnosis had already been established and documented in the patient’s medical record before study inclusion; the present analysis relied on previously documented clinical diagnoses rather than on spirometric adjudication);
2. Age ≥18 years;
3. Permanent residence in the Podlaskie Voivodeship;
4. Ability to complete the questionnaires independently, as assessed by medical staff;
5. Voluntarily provision of informed consent to participate in the study.

The following individuals were excluded from the study:

1. Patients with previously diagnosed severe mental disorders that could affect the reliability of responses (e.g., schizophrenia, bipolar disorder);
2. Patients with cognitive impairments that prevented reliable completion of the questionnaires (e.g., dementia);
3. Patients in a severe somatic condition that prevented safe participation in the study;
4. Patients who did not consent to participate in the study or withdrew their consent at any stage.

### 2.3. Research Tools

#### 2.3.1. Original Survey Questionnaire

The original survey questionnaire, developed for this study, was anonymous and consisted of a total of 11 single- and multiple-choice questions, covering both socio-demographic data (6 questions, including age, gender, marital status, education, place of residence, and financial situation), as well as information on the course of the disease and subjective assessment of COPD symptoms (5 questions, including time since COPD diagnosis, assessment of COPD symptom severity, assessment of the impact of COPD symptoms on daily functioning, COPD treatment methods used, and noticeable side effects of COPD treatment).

#### 2.3.2. Beck Depression Inventory (BDI)

The severity of depressive symptoms was assessed using the Beck Depression Inventory (BDI), developed by Aaron T. Beck et al. in 1961 [21]. In 1996, a revised version of the tool was developed—the BDI-II, which was adjusted to the DSM-IV depression criteria [22].

The scale was translated and validated in Poland in 2009 with its high validity and reliability confirmed [23]. Polish studies reported a high Cronbach’s alpha coefficient (≥0.90).

The questionnaire consists of 21 items, rated on a scale of 0–3, resulting in a total score of 0–63. The following interpretation was adopted in this study:

- 0–8 points—no depressive symptoms;
- 9–18 points—mild depression;
- ≥19 points—moderate/severe depressive symptoms.

Higher scores indicate greater symptom severity.

#### 2.3.3. Hamilton Anxiety Rating Scale (HAM-A)

Anxiety severity was assessed using the Hamilton Anxiety Rating Scale (HAM-A), developed by Max Hamilton in 1959 as a clinical measure of anxiety symptoms [24].

The scale comprises 14 items, rated in a range of 0–4, with a total score of 0–56. The items cover both psychological symptoms (e.g., tension, anxiety) and somatic symptoms (e.g., cardiovascular and respiratory symptoms).

The most commonly used interpretation has been presented below:

- <17 points—mild anxiety;
- 18–24 points—mild/moderate anxiety;
- 25–30 points—moderate/severe anxiety;
- 30 points—very severe anxiety.

The reliability of the HAM-A in international studies is rather high (Cronbach’s alpha between 0.77 and 0.90) [25]. Polish translations of the tool exist and are used in clinical practice, albeit without a single, comprehensive population validation.

In the present study, the HAM-A was completed by members of the research team and practicing physicians working in the Department of Allergology and Internal Medicine of the University Teaching Hospital in Bialystok.

#### 2.3.4. Generalized Anxiety Disorder-7 (GAD-7)

Symptoms of generalized anxiety were evaluated using the Generalized Anxiety Disorder-7 (GAD-7), which was developed in 2006 as a brief screening tool for identifying and measuring anxiety severity [26].

The scale consists of 7 questions, rated on a scale of 0–3, giving a total score of 0–21. The most commonly applied clinical thresholds are as follows:

- 5 points—mild anxiety;
- 10 points—moderate anxiety (a cut-off point with high sensitivity and specificity);
- and 15 points—severe anxiety.

In the original studies, Cronbach’s alpha amounted to 0.92, indicating excellent reliability [18]. In Poland, the scale was validated in 2023, confirming high reliability (α > 0.80) and a unifactorial structure [27].

#### 2.3.5. Athens Insomnia Scale (AIS)

Insomnia symptoms were assessed using the Athens Insomnia Scale (AIS), developed in 2000 based on ICD-10 criteria [28].

The scale comprises 8 items rated on a 0–3 scale, covering both sleep parameters and daytime functioning. The total score ranges from 0 to 24 points.

Scale scores are interpreted as follows:

- Classical cut-off point: ≥6 points,
- In the Polish validation, the optimal cut-off point: ≥8 points [29].

The Polish adaptation [29] demonstrated high reliability (Cronbach’s alpha = 0.90) and excellent temporal stability (ICC = 0.92).

#### 2.3.6. Insomnia Severity Index (ISI)

The severity of subjective sleep difficulties was assessed using the Insomnia Severity Index (ISI) developed in 2001 [30].

The questionnaire contains 7 items, rated on a scale of 0–4, with a total score of 0–28.

Interpretation:

- 0–7 points—no clinically significant difficulties;
- 8–14 points—mild (sub-threshold) insomnia;
- 15–21 points—moderate insomnia;
- 22–28 points—severe insomnia.

The reliability of the ISI in international studies is high (Cronbach’s alpha between 0.80 and 0.90) [31]. Polish publications indicate a high usefulness of the tool in the Polish-speaking population.

#### 2.3.7. Sexual Quality of Life Questionnaire (SQoL)

Sexual quality of life was assessed using:

- The Standardized Sexual Quality of Life Questionnaire—Female (SQoL-F), developed in 2005 [32];
- The Standardized Sexual Quality of Life Questionnaire—Male (SQoL-M), developed in 2012 [33].

SQoL-F contains 18 items and SQoL-M has 11 items rated on a 6-point Likert scale. The raw score, calculated as the sum of points obtained in individual items, was converted to a standardized score on a 0–100 scale according to the authors’ instructions. The conversions were carried out separately for the female (SQoL-F) and male (SQoL-M) versions, taking into account the differences in the design of the tools (including the number of items and the range of possible raw scores), which ultimately allowed for the presentation of results on a comparable scale. In the original studies, reliability amounted to α ≈ 0.90 for both versions [32,33]. The questionnaires were also used in Polish populations, which confirms their usefulness in research concerning the quality of sexual life.

### 2.4. Ethical Considerations

The study was conducted in accordance with the Declaration of Helsinki. The project obtained approval from the Bioethics Committee of the Medical University of Bialystok, Poland (Recommendation No.: APK.002.28.2025 of 23 January 2025). The participants were informed of the anonymity of their data.

### 2.5. Statistical Analysis

Statistical analysis was performed using STATISTICA 13.3 PL (StatSoft Polska, Krakow, Poland), and supplementary analyses were conducted using RStudio 2026.01.1 (R version 4.5.2) (Posit Software, PBC, Boston, MA, USA). Quantitative variables were described using descriptive statistics appropriate to their distribution. The normality of quantitative variables was assessed with the Shapiro–Wilk test and the results are presented in Table 1. Because most outcome variables deviated from normality, non-parametric methods were applied in the primary analyses. Accordingly, Mann–Whitney U tests, Kruskal–Wallis tests, and Spearman’s rank correlations were used. Supplementary analyses were additionally performed. These included age-stratified correlation analyses and multivariable linear models for BDI, HAM-A, GAD-7, AIS, ISI, and SQoL, including hospitalization status, disease duration, and subjective symptom severity. Bonferroni correction was applied where appropriate for repeated testing. Results were considered statistically significant at *p* < 0.05, with adjusted interpretation when multiple comparisons were performed.

## 3. Results

### 3.1. Socio-Demographic Characteristics of the Study Group

In total, 47 patients diagnosed with COPD participated in the analysis. The majority were men (59.6%) and hospitalized patients (76.6%). More than 70% of the study participants were in a relationship, and 61.7% had secondary or post-secondary education. The vast majority rated their financial situation as good (68.1%).

Detailed demographic characteristics are presented in Table 2.

### 3.2. Standardized Scale Scores in the Whole Group

Table 3 portrays full descriptive statistics for the following scales: depression (BDI), generalized anxiety (GAD-7), somatic anxiety (HAM-A), insomnia (AIS, ISI), and sexual quality of life (SQoL).

The median severity of depression symptoms amounted to 16 points, while the medians for the anxiety and insomnia scales indicated clinically significant burden. Sexual satisfaction, assessed using the SQoL summary score, also showed significant variation (Me = 34.12; IQR: 21.82–80.00).

### 3.3. Socio-Demographic Factors and Mental Status

#### 3.3.1. Age

Patient age positively correlated with the severity of all examined mental symptoms—depression (R = 0.53; *p* < 0.001), anxiety (HAM-A: R = 0.51; GAD-7: R = 0.45; *p* < 0.01), and insomnia (AIS: R = 0.42; ISI: R = 0.45; *p* < 0.01). A negative correlation was also found between age and sexual quality of life (SQoL: R = −0.63; *p* < 0.001). However, supplementary age-stratified analyses did not confirm these associations consistently across all age subgroups.

#### 3.3.2. Gender

Women had significantly higher BDI, HAM-A, GAD-7, AIS, and ISI scores (all *p* < 0.01) compared to men. No differences were determined in terms of SQoL (*p* > 0.05).

Sex-related differences were further evaluated using rank-biserial effect sizes and supplementary regression models adjusted for age and disease duration. Women had higher crude scores than men on several psychological measures, and the magnitude of these differences was moderate to large for selected outcomes. For example, the effect size for BDI was rank-biserial correlation = 0.58 (95% CI: 0.31 to 0.76). In adjusted regression models, the effect of sex remained statistically significant for HAM-A and ISI, while for other outcomes the differences were less stable after accounting for age and disease duration.

#### 3.3.3. Marital Status, Place of Residence, and Financial Situation

No significant differences were found in the results of any psychological scales depending on marital status, place of residence, or self-assessed financial situation (all *p* > 0.05).

### 3.4. COPD and Mental Health

#### 3.4.1. Disease Duration

A longer duration of COPD involved higher severity of symptoms of depression, anxiety, and insomnia (H = 22.28–23.59; all *p* < 0.001). Simultaneously, a decrease in the quality of sexual life was observed with disease duration (H = 24.86; *p* < 0.001).

#### 3.4.2. Subjective Severity of COPD Symptoms

The subjective assessment of symptom severity exhibited strong positive correlation with scores in all psychological scales (R = 0.72–0.75; *p* < 0.001) and negatively with the quality of sexual life (SQoL: R = −0.75; *p* < 0.001).

#### 3.4.3. Daily Functioning

Patients reporting significant limitations in daily functioning achieved significantly higher BDI, HAM-A, GAD-7, AIS, and ISI scores, and lower SQoL scores (H = 32.46; *p* < 0.001).

#### 3.4.4. Treatment Location

Hospitalized patients scored significantly higher on all psychological scales compared to outpatients (*p* < 0.001). For GAD-7, the median was 10.0 points in inpatients and 1.0 point in outpatients, while for SQoL, the median was 31.11 versus 75.56 points, respectively. Full descriptive statistics for both groups are presented in Table 4.

However, because of the marked imbalance between groups and the possibility that hospitalization reflected greater acute symptom burden, supplementary adjusted analyses were performed. These models indicated that subjective symptom severity remained the most consistent predictor across outcomes, whereas the independent effect of hospitalization status was attenuated after adjustment. Supplementary sensitivity analyses confirmed that the observed between-group pattern remained stable despite the marked group imbalance and floor effects.

### 3.5. Co-Occurrence of Psychiatric Symptoms

Outstandingly strong correlations were observed between symptoms of depression, anxiety, and insomnia. Depressive symptoms correlated with both generalized anxiety symptoms (R = 0.86; *p* < 0.001) and somatic anxiety symptoms (R = 0.90; *p* < 0.001). Anxiety symptoms were also strongly associated with sleep disorders (AIS/ISI: R = 0.80–0.91; *p* < 0.001).

### 3.6. Sexual Quality of Life and Mental Health

Significant negative correlations were found between the SQoL score and:

- Depression (BDI: R = −0.74; *p* < 0.001);
- Anxiety (HAM-A: R = −0.75; GAD-7: R = −0.64; *p* < 0.001);
- Insomnia (AIS: R = −0.66; ISI: R = −0.69; *p* < 0.001).

## 4. Discussion

The study found a high psychological burden in patients with COPD, including increased levels of depression, anxiety, and sleep disorders and decreased sexual satisfaction. These results are consistent with the concept of COPD as a multidimensional disease in which respiratory symptoms and functional limitations coexist with psychological and social consequences, as emphasized by reviews and analyses of research needs in this area [4,8,9]. Notwithstanding the above, the clinical picture indicates that COPD is frequently associated with the co-occurrence of several conditions, including depression, anxiety, insomnia, and sexual dysfunction, which may together constitute a self-reinforcing constellation of interrelated difficulties rather than a set of independent, parallel disorders.

### 4.1. The Role of Course Severity and Hospitalization

The significantly poorer psychological outcomes observed in the hospitalized group of patients in our study are consistent with the interpretation that lower clinical stability and higher symptom burden increase the risk of depression, anxiety, and insomnia. Several literature sources emphasize that a greater subjective symptom burden and increased dyspnea contribute to mental disorders, while the coexistence of depression and anxiety may increase the risk of further adverse clinical events (including through poorer self-care and reduced activity) [4,8,9,34]. In this context, it is worth interpreting hospitalization not only as a socio-demographic variable, but also as a marker of an episode of clinical deterioration and increased illness-related stress.

These findings should be interpreted with caution due to the relatively small number of women in the study and the limited stability of some sex-related effects after adjustment for age and disease duration.

Additionally, the observed gender differences are consistent with the results obtained by Di Marco et al., who indicated the role of gender and disease severity in shaping depressive and anxiety symptoms in COPD [6]. Psychological factors (such as health anxiety, a sense of loss of control) and social factors (care-giving role, symptom reporting style, cultural norms) can modulate the way the disease and its consequences are experienced [6].

Hospitalization may be strongly associated with psychological burden for several, partly overlapping, reasons. Patients treated in inpatient settings are more likely to experience greater acute symptom burden, functional limitation, uncertainty regarding their health status, and disruption of everyday routines, all of which may intensify depressive symptoms, anxiety, and sleep difficulties [4,11]. At the same time, poorer mental health may itself aggravate the clinical experience of COPD by increasing distress related to dyspnea, reducing coping capacity, and limiting engagement with treatment recommendations [4,8]. Therefore, the relationship between respiratory burden and psychological functioning is likely to be bidirectional [8,11]. However, in the present study, this issue should be interpreted with caution because no objective COPD severity measures were available and the cross-sectional design does not allow conclusions regarding temporal sequence or causality. The supplementary adjusted analyses suggest that subjective symptom severity was more consistently associated with psychological outcomes than hospitalization status alone, which supports a more careful interpretation of inpatient–outpatient differences.

### 4.2. Depression and Anxiety as Part of the Clinical Presentation of COPD

Our research results have confirmed that depression and anxiety are among the most frequently observed disorders in COPD; they are also clinically significant. A meta-analysis by Atlantis et al. indicates a bidirectional relationship: on the one hand, COPD is associated with an increased risk of depression and anxiety, and on the other, clinically significant depressive/anxiety symptoms correlate with a worse course of COPD and adverse outcomes [9]. Similar conclusions are drawn from the reviews by Yohannes and Alexopoulos [4] and Maurer et al. [15], who emphasize that psychiatric comorbidity affects patients’ quality of life, functioning, and disease prognosis. In our study, strong correlations between the severity of depression and anxiety and other dimensions of functioning (including sleep and sexuality) support the interpretation that mental state is an integral component of the clinical picture of COPD, not simply a reaction to the disease.

A mechanistic explanation for the observed relationships is important. Literature sources often discuss the “vicious cycle” model of dyspnea and anxiety: dyspnea increases tension and somatic alertness, which, in turn, intensifies anxiety and exercise avoidance, leading to de-condition, decreased exercise tolerance, and a renewed increase in dyspnea [8]. Within this approach, increased anxiety may exacerbate respiratory symptoms and functional limitations, while simultaneously limiting the effectiveness of recommendations (e.g., activation, rehabilitation). What is more, it is emphasized that depression can reduce motivation for activity, increase the feeling of helplessness, and reduce the ability to self-care, which, in the case of chronic disease, has a direct impact on the course and control of the symptoms [4,8].

At the biological level, it is increasingly pointed out that the COPD–depression relationship may be partially exacerbated by systemic inflammation. Lu et al. demonstrated associations between elevated inflammatory markers (including IL-6, CRP) and increased frequency of depressive symptoms as well as increased obstructive symptoms [35]. This perspective is interpretatively important as it suggests that some depressive symptoms in COPD may result not only from psychosocial burdens, but also from biological processes accompanying the disease [35].

### 4.3. Sleep Disorders as a Significant Clinical Issue

In our study, sleep disorders were common and strongly associated with depression and anxiety. This is consistent with the results found by Saaresranta et al. [7], who demonstrated a relationship between sleep quality and daytime symptoms and the quality of functioning in COPD. A review performed by Budhiraja et al. [36] revealed the multifactorial background of sleep disorders in this population: nocturnal respiratory symptoms, hypoxemia/hypercapnia, cough, medication effects, and psychological factors (anxiety, stress). Importantly, there is growing evidence that disturbed sleep may have prognostic significance [36]. Omachi et al. [37] demonstrated that sleep disturbances in patients with COPD are longitudinally associated with adverse outcomes, including a higher risk of death and worse COPD endpoints. This finding reinforces the practical conclusion that the assessment of insomnia and sleep quality should be considered a clinically relevant element, potentially influencing the course of the disease, and not solely as a side effect of dyspnea.

The coexistence of COPD with obstructive sleep apnea (the so-called overlap syndrome) is also a crucial issue. McNicholas highlights the overlap of patho-physiological mechanisms and cardiovascular risk in this group [38]. The report by Marin et al. [39] is particularly specific here: overlap syndrome was associated with an increased risk of death and hospitalization due to COPD exacerbations, while CPAP (Continuous Positive Airway Pressure) treatment was associated with improved survival and reduced hospitalization [39]. In the context of our results, this is of interpretive significance: some insomnia cases, reduced sleep quality, or daytime sleepiness may result from sleep-disordered breathing, not just from mental stress, which justifies the diagnosis of obstructive sleep apnea (OSA) in patients with severe sleep disorders and high clinical risk [39].

The relationship between sleep and depression/anxiety is also reciprocal. It has been shown that insomnia contributes to the perpetuation of depressive and anxiety symptoms, while anxiety and depression, on the other hand, worsen sleep quality. In clinical trials, the co-occurrence of these problems has been observed in patients with COPD [40]. This suggests that sleep-focused interventions (e.g., behavioural interventions, sleep hygiene, sleep disordered breathing diagnostics) may indirectly reduce the severity of anxiety and depression, thus improving functioning in chronic disease [15,40].

### 4.4. Sexual Satisfaction and Patients’ Mental State

The results of our study (reduced sexual satisfaction and its strong association with depression, anxiety, and insomnia) are consistent with the literature. Zysman et al. [41] demonstrated that the quality of sexual life in patients with COPD is significantly reduced and emphasize that sexual dysfunctions are common and tend to significantly impact well-being, yet remain neglected in clinical practice. A systematic review and meta-analysis conducted by Farver-Vestergaard et al. [14] indicated a significant deterioration in sexual health in COPD and the association of these problems with quality of life and symptom burdens. These results correspond with our own observations, in which sexual satisfaction remained strongly associated with mental state and sleep. However, these relationships should be interpreted as associative rather than causal, because the cross-sectional design of the present study does not allow conclusions regarding temporal order or directionality. Longitudinal studies are needed to clarify whether reduced sexual satisfaction precedes, follows, or co-develops with depressive symptoms, anxiety, and insomnia.

Clinical communication remains a critically important aspect. Kaptein et al. [42] indicated that patients with chronic respiratory diseases rarely discuss sexuality with medical staff, despite the importance of this topic for their quality of life. Rubio-Rask et al. [43] also emphasize that the area of sexual health in COPD requires active disclosure in clinical contact, and that designed educational materials can support patient–medical staff communication. This suggests that the sexual sphere should be considered in the assessment of a COPD patient, especially when symptoms of depression, anxiety, or insomnia coexist [16,41,42,43,44].

### 4.5. Age, Disease Duration, and Deterioration of Mental Health

In our study, older age and longer disease duration were substantially associated with higher severity of symptoms of depression, anxiety, and sleep disorders, as well as with lower quality of sexual life. These results are consistent with literature reports emphasizing that the chronicity of the disease, the increasing functional limitations, and a sense of dependence on the environment increase the risk of developing mental disorders [4,40].

Although age in the full sample was associated with worse psychological outcomes and lower sexual quality of life, these associations were not consistently reproduced in age-stratified analyses. This suggests that age effects in the present pilot sample may partly reflect sample structure and limited subgroup size rather than a stable linear gradient across all age categories. Therefore, the age-related findings should be interpreted cautiously and verified in larger samples.

### 4.6. Comorbidity of Mental Disorders—A Multidimensional Burden

The strong coexistence of depression, anxiety, insomnia, and decreased sexual satisfaction in our study suggests a mechanism in which (1) dyspnea and functional limitations increase stress and anxiety, (2) anxiety and depression increase exercise avoidance and worsen sleep, (3) poor sleep exacerbates affective symptoms and reduces symptom tolerance, and (4) the accumulation of these factors may reduce well-being and quality of life, including intimate aspects [8,14,36,37,44,45]. This approach supports the conclusion that interdisciplinary care is necessary and that mental health and sleep assessment should be included in the standard evaluation of COPD patients [1,4,8].

The very strong correlations observed among depression, anxiety, and insomnia may reflect not only genuine co-occurrence of symptoms, but also conceptual overlap between constructs and common-method variance, because several self-report instruments were administered simultaneously in the same clinical context. In addition, the HAM-A includes somatic items that may overlap with COPD-related symptom burden. For this reason, the magnitude of these associations should not be interpreted as evidence of directionality or causality.

### 4.7. Study Limitations

This study has several limitations. First, its cross-sectional design precludes causal inference. Second, the sample was relatively small and recruited from a single centre, which limits generalizability. Moreover, the sample size should be interpreted in the context of the pilot nature of the study. Post hoc sensitivity considerations indicated that the present sample was mainly informative for moderate-to-large effects, whereas smaller associations may not have been detectable with sufficient statistical power. Therefore, the findings should be regarded as exploratory and hypothesis-generating rather than confirmatory. Third, there was a marked imbalance between inpatients and outpatients, and the outpatient subgroup was small, which reduces the stability of between-group comparisons. Fourth, no objective COPD severity measures, such as FEV1, GOLD stage, mMRC, CAT score, exacerbation history, or oxygen therapy use, were available; therefore, the results should not be interpreted as directly reflecting objective clinical severity. Fifth, the study relied predominantly on self-report instruments, which may increase the risk of response bias and common-method variance. Sixth, structured comorbidity data were not available for inferential adjustment. Finally, although the project had a pilot character, formal feasibility outcomes were not recorded in sufficient detail for separate reporting.

## 5. Conclusions

In this pilot cross-sectional study, patients with COPD showed a considerable within-sample burden of depressive symptoms, anxiety, insomnia, and reduced sexual quality of life. The examined psychological outcomes were particularly associated with subjective symptom burden. Crude differences between inpatients and outpatients should be interpreted cautiously, as analyses indicated substantial confounding by symptom severity. Given the small single-center sample, group imbalance, and lack of objective COPD severity measures, the findings should be regarded as exploratory and require confirmation in larger multicenter studies.

## Figures and Tables

**Table 1:** Shapiro–Wilk normality test results for quantitative variables.

Variable	W	*p*
Age	0.95933	0.101
BDI	0.84686	<0.001
HAM-A	0.87605	<0.001
GAD-7	0.95355	0.06
AIS	0.90881	0.001
ISI	0.93402	0.011
SQoL (standardized)	0.86754	<0.001

Abbreviations: BDI—Beck Depression Inventory, HAM-A—Hamilton Anxiety Rating Scale, GAD-7—Generalized Anxiety Disorder-7, AIS—Athens Insomnia Scale, ISI—Insomnia Severity Index, SQoL—Sexual Quality of Life Questionnaire, W—Shapiro–Wilk test, *p*—*p*-value.

**Table 2:** Socio-demographic characteristics of the study group (*n* = 47).

Variable	Categories	*n* (%)
Gender	Female	19 (40.4%)
	Male	28 (59.6%)
Treatment location	Outpatients	11 (23.4%)
	Inpatients	36 (76.6%)
Marital status	In a relationship	33 (70.2%)
	Others	14 (29.8%)
Education	Vocational/primary	6 (12.8%)
	Secondary	15 (31.9%)
	Post-secondary	14 (29.8%)
	Higher	10 (21.3%)
Place of residence	Countryside	10 (21.3%)
	Small town	8 (17.0%)
	Medium town/city	19 (40.4%)
	Large city	10 (21.3%)
Material situation	Moderate	15 (31.9%)
	Good	32 (68.1%)

**Table 3:** Results of the research scales in the study group (*n* = 47).

Scale	M	SD	Me	Q_1_	Q_3_	Min.	Max.
BDI	19.89	16.96	16	2.50	34.50	2	55
HAM-A	23.49	17.03	27	6.50	40.00	0	50
GAD-7	8.06	5.83	8	2.50	12.50	0	21
AIS	9.19	6.38	9	8.00	13.00	0	29
ISI	13.79	7.87	14	9.50	18.50	0	28
SQoL (total)	51.51	30.68	34.12	21.82	80.00	12.73	100

Abbreviations: M—arithmetic mean, SD—standard deviation, Me—median, Q_1_—lower quartile, Q_3_—upper quartile, Min.—minimum, Max.—maximum, BDI—Beck Depression Inventory, HAM-A—Hamilton Anxiety Rating Scale, GAD-7—Generalized Anxiety Disorder-7, AIS—Athens Insomnia Scale, ISI—Insomnia Severity Index, SQoL—Sexual Quality of Life Questionnaire.

**Table 4:** Differences between outpatients (*n* = 11) and inpatients (*n* = 36).

Scale	Group	M	SD	Me	Q_1_	Q_3_	Min.	Max.	*p*
BDI	Outpatients	2.18	0.40	2.0	2.0	2.0	2	3	*p* < 0.001
	Inpatients	25.31	15.78	30.5	7.75	36.25	2	55	
HAM-A	Outpatients	4.45	6.23	2.0	0.0	21.0	0	21	*p* < 0.001
	Inpatients	29.31	14.88	36.0	10.0	39.25	0	50	
GAD-7	Outpatients	1.82	2.52	1.0	0.0	2.5	0.0	7.0	*p* < 0.001
	Inpatients	9.97	5.19	10.0	6.75	14.0	0.0	21.0	
AIS	Outpatients	3.72	5.34	1.0	0.0	9.0	0	14	*p* < 0.001
	Inpatients	10.86	5.64	12.0	8.0	14.0	0	29	
ISI	Outpatients	5.36	5.71	4.0	0.0	9.5	0	14	*p* < 0.001
	Inpatients	16.36	6.55	17.0	14.0	21.0	0	28	
SQoL (standardized)	Outpatients	81.63	17.32	75.56	71.11	100.0	52.73	100.0	*p* < 0.001
	Inpatients	40.29	26.92	31.11	20.0	61.14	12.73	100.0	

Abbreviations: M—arithmetic mean, SD—standard deviation, Me—median, Q_1_—lower quartile, Q_3_—upper quartile, Min.—minimum, Max.—maximum, BDI—Beck Depression Inventory, HAM-A—Hamilton Anxiety Rating Scale, GAD-7—Generalized Anxiety Disorder-7, AIS—Athens Insomnia Scale, ISI—Insomnia Severity Index, SQoL—Sexual Quality of Life Questionnaire.

## Data Availability

The raw data supporting the conclusions of this article will be made available by the authors on request.
